# Effectiveness of a Postpartum Text Message Program (Essential Coaching for Every Mother) on Maternal Psychosocial Outcomes: Protocol for a Randomized Controlled Trial

**DOI:** 10.2196/27138

**Published:** 2021-03-25

**Authors:** Justine Dol, Megan Aston, Douglas McMillan, Gail Tomblin Murphy, Marsha Campbell-Yeo

**Affiliations:** 1 Faculty of Health Dalhousie University Halifax, NS Canada; 2 School of Nursing Faculty of Health Dalhousie University Halifax, NS Canada; 3 Department of Pediatrics Faculty of Medicine Dalhousie University Halifax, NS Canada; 4 Nova Scotia Health Halifax, NS Canada

**Keywords:** text message, mobile health, postpartum education, self-efficacy, social support, postpartum anxiety, postpartum depression

## Abstract

**Background:**

Women experience changes both physically and psychologically during their transition to motherhood. The postnatal period is a critical time for women to develop maternal self-efficacy. Mobile health interventions may offer a way to reach women during this critical period to offer support and information. Essential Coaching for Every Mother is a text message program that seeks to educate and support women during the first 6 weeks postpartum.

**Objective:**

The primary effectiveness objective is to compare the effectiveness of the Essential Coaching for Every Mother program on maternal psychosocial outcomes (self-efficacy, social support, postpartum depression, and postpartum anxiety) immediately after the intervention and 6 months postpartum, collectively as well as stratified by parity. The primary implementation objective is to evaluate the implementation extent and quality of the Essential Coaching for Every Mother program.

**Methods:**

This will be a hybrid type 1 effectiveness-implementation randomized controlled trial. A total of 140 mothers-to-be or new mothers from Nova Scotia will be recruited and randomized to the intervention or control arm, stratified by parity. The intervention arm will receive the Essential Coaching for Every Mother program, which consists of 53 messages sent twice a day for the first 2 weeks and daily for weeks 3 through 6. The control group will receive usual care. Messages are personalized based on the infant’s age and the woman’s self-selected preference for breastfeeding or formula feeding and tailored with the infant’s name and gender. Women can enroll in the program if they are ≥37 weeks pregnant or within 10 days postpartum, with the first message designed to be sent on the second evening after birth. The actual number of messages received will vary based on the timing of enrollment and the infant’s date of birth. Participants will complete questionnaires assessing self-efficacy, social support, and postpartum depression and anxiety at baseline (enrollment after birth) and 6 weeks (postintervention) and 6 months postpartum. Implementation data will be collected throughout the trial, and evaluation feedback will be collected at 6 weeks from women who received the intervention.

**Results:**

Recruitment for this study started on January 5, 2021, and is currently ongoing, with an anticipated date of recruitment completion of January 2022.

**Conclusions:**

This study will assess the effectiveness of a postpartum text message program to improve maternal self-efficacy and social support while decreasing postpartum depression and anxiety. It will also shed light on the implementation effectiveness of the program.

**Trial Registration:**

ClinicalTrials.gov NCT04730570; https://clinicaltrials.gov/ct2/show/NCT04730570

**International Registered Report Identifier (IRRID):**

DERR1-10.2196/27138

## Introduction

### Background

During the postpartum period, women require support and information to ensure a smooth transition and adjustment to motherhood, which is reflected in both the World Health Organization’s postnatal guidelines [1] and the Public Health Agency of Canada’s maternity and postnatal care guidelines [2]. Worldwide, the postnatal period is often neglected in the provision of quality care [1,3]. During pregnancy, women are regularly monitored, especially during the third trimester with weekly or biweekly appointments, yet after they give birth the frequency of monitoring is significantly reduced to only once or twice in the first year, typically first around 6 weeks postpartum [4,5]. The amount and type of postpartum care that women receive for themselves and their infants depend on whether they are being followed by doctors or midwives, they are considered to have high needs, or they have identified health concerns for themselves or their infants [6].

Although the postnatal period provides an opportunity to educate and support new mothers, many women have reported not having enough information to help them in their transition to motherhood, resulting in challenges to their psychosocial adjustment [7,8]. While the postpartum period may be uncomplicated for some women, evidence suggests that others may struggle with low self-efficacy [9], feel isolated or struggle with low social support [9,10], and experience symptoms of anxiety and depression [10,11]. There is a need to develop and evaluate innovative approaches to educate and support women during this period to ensure that maternal psychosocial health needs are met and concerns are addressed in a timely manner. Evidence suggests that women are turning to online and mobile apps for sources of information during the perinatal period [12,13]. Therefore, there is an opportunity to develop a standardized, evidence-based mobile health (mHealth) intervention to address known concerns around maternal psychosocial adjustment to enhance their feelings of self-efficacy and social support while decreasing levels of postpartum anxiety and depression.

While postpartum mobile apps are also growing in prevalence, a recent systematic review identified 48 mobile apps of varying quality [14]. Also, mobile apps require access to data plans, rely on push notifications that may not be enabled, and are time consuming to build. On the other hand, one aspect of mHealth that shows significant promise is text messaging because of its cost-effectiveness, broad reach, and simplicity of use. There is some evidence that text messaging programs can enhance maternal and newborn outcomes and engage mothers during the postnatal period [15,16]. Existing postpartum text message programs focus on various postpartum topics—text4baby aims to improve perinatal health for women and their infants [17], MobileMums is focused on enhancing physical activity [18], and MumBubConnect’s goal is to promote breastfeeding [19]—yet no postpartum text message programs are currently available in Canada. The provision of timely health information can influence behaviors through repeated and relevant health messages [20]. Text messages can be sent, saved, and retrieved at any time [21], are able to be personalized and tailored, and can be provided through one- or two-way communication [22]. Therefore, a text message program was designed with low barriers to entry, low cost for participation, and ease of development and modification.

Our team developed Essential Coaching for Every Mother, an evidence-based postpartum text message program, with the goal of improving mothers’ access to information about caring for themselves and their newborn during the immediate 6-week period after birth [23]. The program was developed through iterative testing with mothers and postpartum health care providers [23] and showed preliminary effectiveness and uptake during a pilot pre-post intervention study offered during COVID-19 [24].

### Effectiveness Objectives

The primary effectiveness objective of this study is to compare the effectiveness of the Essential Coaching for Every Mother program to standard care on self-efficacy measured immediately after the intervention. The secondary objectives are (1) to compare the effectiveness of the Essential Coaching for Every Mother program to standard care on social support, postpartum anxiety, and postpartum depression immediately after the intervention; (2) to determine whether the effect on any outcomes differs based on parity (multiparous or primiparous); and (3) to compare any long-term impacts of the Essential Coaching for Every Mother program on the above outcomes at 6 months postpartum.

### Effectiveness Hypotheses

The primary hypothesis is that the Essential Coaching for Every Mother program will result in higher self-efficacy for participants who receive the intervention compared with standard care. The secondary hypotheses are that (1) Essential Coaching for Every Mother will result in higher levels of social support, lower levels of postpartum anxiety, and lower levels of postpartum depression for those who receive the intervention compared with standard care; (2) there will be no difference based on parity on any of the psychosocial outcomes; and (3) changes in psychosocial outcomes will be maintained over time.

### Implementation Objective

The implementation objective is to evaluate the implementation extent (ie, enrollment rate and timing, withdrawal rate, number of messages received) and quality (ie, satisfaction, barriers, suggestions for improvement) of the Essential Coaching for Every Mother program.

## Methods

### Study Design

This study will be a 2-group, stratified, parallel-arm, hybrid type 1 effectiveness-implementation randomized controlled trial. Through the use of an effectiveness-implementation design, it is possible to evaluate intervention effectiveness while simultaneously evaluating real-world implementation to guide future development and revisions [25]. The intervention will be compared with standard care that is currently being provided across Nova Scotia, stratified by parity.

### Ethics Approval

The study has been approved by the IWK Health Research Ethics Board (#1024984) and the Nova Scotia Health Research Ethics Board (#1026534) and is registered with the clinicaltrials.gov Protocol Registration System (NCT04730570). The CONSORT-EHEALTH (Consolidated Standards of Reporting Trials of Electronic and Mobile HEalth Applications and onLine TeleHealth) checklist [26] will be followed to improve the reporting as an eHealth intervention trial. In reporting the process evaluation, the Template for Intervention Description and Replication (TIDieR) checklist and guideline will be used [27,28]. The TIDieR checklist offers a standardized way to describe the implementation and document any deviations that arise, which allows for not only replication but also clear reporting for comparison of interventions [27].

### Setting

This study is being conducted across Nova Scotia, Canada. In 2017, Nova Scotia had a total of 8197 live births [29]. Currently in Nova Scotia and across Canada broadly, there is no standardization of delivery of postpartum care within or between provinces [6]. Thus, there is a need for interventions targeting the postnatal period to enhance the postpartum experience for mothers in Nova Scotia.

### Inclusion Criteria

Participants will be recruited antenatally and postnatally. To enroll antenatally, participants must (1) be at least 37 weeks pregnant; (2) have daily access to a mobile phone with text messaging capabilities; (3) be ≥18 years of age; (4) live and give birth in Nova Scotia; and (5) speak and read English. To enroll postnatally, participants must (1) have an infant younger than 10 days of age; (2) have daily access to a mobile phone with text messaging capabilities; (3) be ≥18 years of age; (4) live and gave birth in Nova Scotia; and (5) speak and read English.

Participants will be excluded if (1) their newborn dies or is expected to die prior to leaving the hospital; (2) they have no access to a mobile phone, either personal or shared; (3) they are unwilling to receive text messages; (4) they decline to participate or withdraw; or (5) they previously participated in the development or feasibility phase of this project.

### Recruitment

A multipronged approach will be used to recruit women who are at least 37 weeks pregnant or within 10 days postpartum. All recruitment will occur remotely with no in-person promotion. The primary methods will be social media advertisements (eg, posting on relevant parent groups on Facebook, paid advertisements on Facebook) as well as study posters across the province (eg, on the Family Newborn Unit at IWK Health, in perinatal centers, in family medical offices, etc). Additional sources of outreach that may be used include promotion through public health, family resource centers, media outreach, posters in public places that pregnant and new parents frequent, and word of mouth.

While the text messages are one-way only, if a participant responds to a message with a question for clarification, the principal investigator or research team member may respond to provide clarification only. For example, if a participant asks if they will receive reminder messages, a brief message will be provided confirming that reminder messages will be sent. If there are requests for advice on their care or that of their baby, mothers will be referred back to their health care provider through a standardized message sent if a participant responds unexpectedly during the program.

### Screening and Baseline

Interested participants will contact the research team via text message to complete the automatic remote eligibility screening process. All participants will receive a welcome message as well as details about the study and what the study will entail. During the antenatal recruitment process, the woman’s phone number, first name, and due date will be collected via TextIt [30]. This information is required to ensure that follow-up messages are sent to the woman via her mobile device based on her due date.

During the antenatal screening process, participants will be screened for eligibility, and eligible participants will be informed of the need to send a text within 10 days after giving birth to be enrolled in the study. Women who have not yet given birth are sent messages at 39 weeks, 40 weeks, 41 weeks, and 42 weeks or until enrolled or withdrawn to remind them of the need to text the word “delivered” after giving birth. Participants who initiate contact before 37 weeks will be asked if they want to be sent follow-up messages once they reach 37 weeks until the sample size is reached.

Once a woman gives birth or enrolls after giving birth, she will be asked screening questions to ensure eligibility and determine parity for stratification. Additional details including the newborn’s name, preferred gender pronoun for the infant, infant’s date of birth, and parity will be collected at that time. Once stratified, participants will be randomized and informed of their group allocation. Participants who are randomized to the intervention group will be asked their preference on breastfeeding and formula feeding messages. All participants will be asked to complete the consent form and baseline survey. To remind participants to complete the survey, reminder text messages will be sent via TextIt every other day for 2 weeks (a maximum of 6 total messages) or until the survey is completed. An email will be sent as a final reminder to participants who have not yet completed the survey after day 14. If an email address was not provided, an expanded text message will be sent on day 14 instead. Participants will also be informed that they may message “STOP” to withdraw from the study at any time. If a participant withdraws from the study, they will not receive any further messages nor will they be asked to complete any future surveys. Participants who complete the baseline survey will receive an electronic gift card worth Can $15 (US $11.97).

### Randomization

Using a 1:1 allocation, participants will be randomized into either the intervention group or the control group using the “split randomly” function within the TextIt platform [30]. This function randomly passes contacts through approximately equally distributed pathways in the flow and will allocate them to receive either the intervention or the control condition. Randomization will occur after a participant has given birth. Participants will be stratified into 2 groups based on parity (primiparous and multiparous) prior to randomization.

### Follow-Up Assessments

At both 6 weeks and 6 months postpartum, all participants in both groups will be sent a text message asking them to complete a follow-up survey. Participants will be asked to provide consent, and once consent is given, they will start the survey. Participants who complete the follow-up surveys will receive an electronic gift card worth Can $15 (US $11.97) for each survey. To remind participants to complete the survey, reminder text messages will be sent via TextIt every other day for 2 weeks (a maximum of 6 total messages) or until the survey is completed. An email will be sent as a final reminder to participants who have not yet completed the survey after day 14. If an email address was not provided, an expanded text message will be sent on day 14 instead.

### Blinding

Because of the nature of the Essential Coaching for Every Mother program, blinding of participants is not possible, and therefore participants will be informed of their group allocation. To minimize personnel blinding related to data collection, no hospital staff will be informed of participants’ involvement in the study. This is possible because Essential Coaching for Every Mother is sent to the participant’s personal cell phone, with no in-person component to the intervention. The lead researcher will be aware of participants’ allocations and will be responsible for summarizing data but will not be involved in the randomization procedure, which will occur directly in the TextIt platform. Additionally, there will be no interaction between the participants and anyone on the research team because everything occurs remotely via a previously set up process, reducing any risk of personnel or outcome assessor bias.

### Sample Size

The sample size was calculated based on the primary outcome of self-efficacy using the findings from our feasibility study [24], which showed a mean difference in self-efficacy scores between baseline and 6-week follow-up of 3.78 (SD 4.5). Therefore, with stratification by parity and a moderate intraclass correlation of ρ=0.2, a sample size of 30 participants per cluster and 60 participants per group (120 total participants) is required to be powered for analysis at the level of parity. In the feasibility study, there was an incompletion rate of 15.5%; thus, the estimated sample size will be increased by 15% to 69 participants per group, rounded up to 70 participants. In total, 140 participants will be enrolled in the study.

### Program Content and Design

#### Text Messages

Essential Coaching for Every Mother consists of 53 standardized text messages that provide evidence-based information related to caring for a newborn and maternal mental health that all new mothers should know. Messages will be sent to participants from birth to 6 weeks postpartum, with 2 messages per day in the first 2 weeks and a daily message in weeks 3 through 6. The following topics are covered in the messages: postpartum anxiety, postpartum depression, maternal self-care, postnatal follow-up, breastfeeding or formula feeding, feeding, infant concerns, cord care, well-baby care, normal development, crying, and safe sleep. Women who receive the messages will be able to self-select breastfeeding or formula feeding messages. The TextIt platform will be used to program and schedule the messages [30], and Twilio will be used as the gateway service that offers virtual phone numbers and short codes that send and receive messages on behalf of the TextIt account [31].

#### Intervention Group

Participants in the intervention group will receive the standard in-person care in the hospital in which they give birth. After enrollment into the study, participants will start receiving messages until 6 weeks postpartum. The messages will be sent automatically based on the age of the infant. Participants in the intervention group will receive Essential Coaching for Every Mother as early as the evening of the second day after giving birth. This could be as early as 17 hours after birth (if a woman delivered at 11:59 the night before) or as late as 41 hours (if a woman delivered at midnight). If a participant enrolls after the messages are designed to start, they will start the message flow based on when they gave birth. Messages are sent at 10 AM and 5 PM during the first 2 weeks and at 10 AM daily after that. This time was recommended by both women and health care providers during the development of the intervention [23].

#### Control Group

Participants in the control group will receive the standard in-person care in the hospital in which they give birth. Women in the control group will not receive any study text messages but are not limited in their ability to seek additional postpartum support outside of the study. Data will be collected for both groups regarding postpartum support sought.

### Study Measures

**Figure 1:**
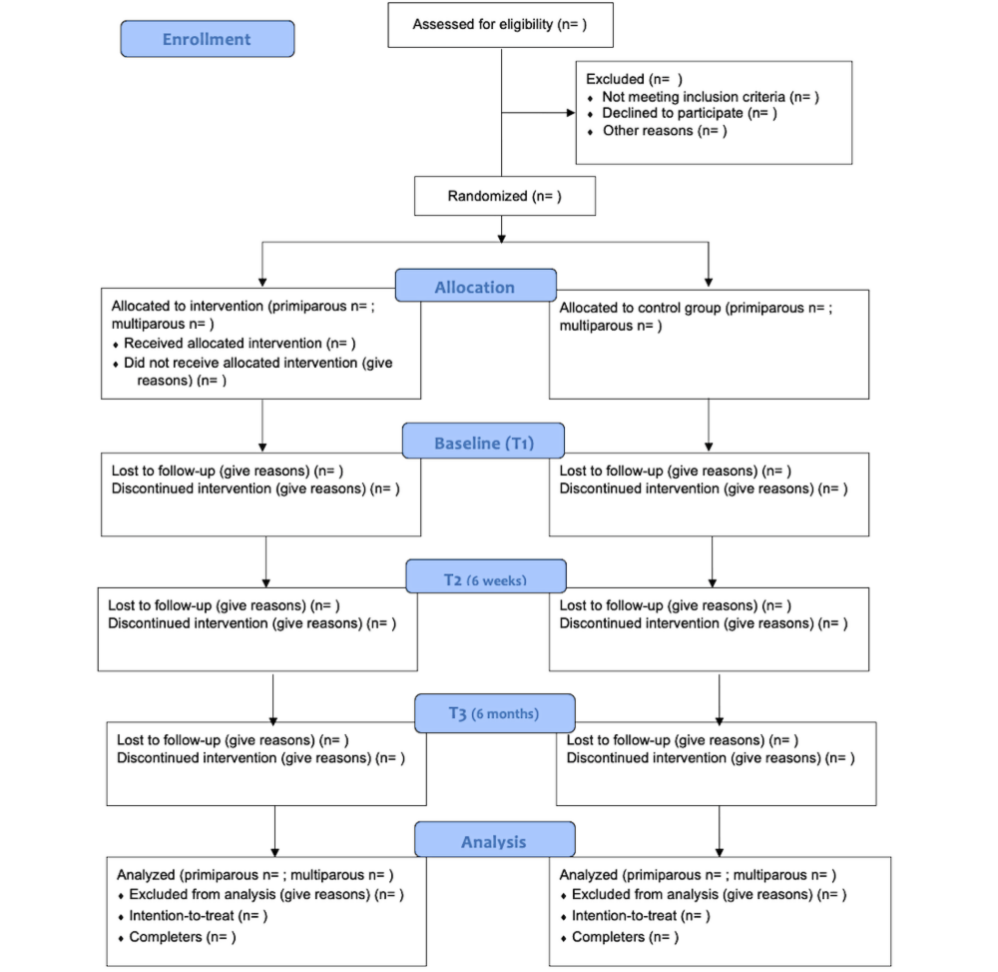
CONSORT (Consolidated Standards of Reporting Trials) flow diagram.

The same data will be collected from all participants, regardless of their group allocation, via self-reported online surveys through Research Electronic Data Capture (REDCap) hosted at IWK Health. Upon enrollment and at 6 weeks and 6 months postpartum, all participants will be sent a text message with a link to a REDCap survey. Background demographics will be collected at baseline on outcomes such as maternal age, occupation, marital status, and education level. At 6 weeks, a follow-up survey will be conducted to collect data on whether mothers had any postnatal contacts for themselves or for their infant, health concerns, health information seeking, infant outcomes, the birth journey (including any intensive care stays), and infant feeding behavior. At 6 months, information on postnatal care and infant feeding behavior will be collected. Implementation data will be collected throughout the trial, and evaluation feedback will be collected at 6 weeks from women who received the intervention. Table 1 provides details of the data collected at each of the outcome assessment time points, and Figure 1 outlines the CONSORT flow diagram.

**Table 1:** Data collected at the study outcome assessment time points.

Data collected			Enrollment		Baseline (T1)		6-week follow-up (T2)		6-month follow-up (T3)
**Enrollment**									
	Eligibility screen	✓							
	Informed consent			✓		✓		✓	
	Randomization	✓							
**Assessments**									
	Demographics			✓					
	Self-efficacy			✓		✓		✓	
	Social support			✓		✓		✓	
	Postpartum anxiety			✓		✓		✓	
	Postpartum depression			✓		✓		✓	
	Postpartum care and adjustment					✓		✓	
	Delivery experience					✓			
	Feeding experience					✓		✓	
	Health information seeking					✓			
**Implementation**									
	Quality					✓			
	Extent	✓		✓		✓			

### Effectiveness Outcomes

The primary outcome of self-efficacy will be measured using the Karitane Parenting Confidence Scale (KPCS) [32]. This 15-item scale assesses the perceived self-efficacy of mothers of infants up to 12 months of age and has acceptable internal consistency (Cronbach α=.81) and test-retest reliability (*r*=0.88) [32]. A cutoff score of ≤39 (out of a possible 45) was determined to indicate a clinically low level of perceived parenting self-efficacy [32]. Additionally, a reliable change index of ≥6 was considered a significant change in their level of parental self-efficacy. Therefore, parents can be considered to have a high level of parenting self-efficacy if they either have a score above 39 or improve their score by 6 points, while still less than 39 [32]. This measurement will be collected at baseline and 6 weeks and 6 months postpartum.

Social support will be measured using the Multidimensional Scale of Perceived Social Support (MSPSS) [33]. This scale provides a measurement of how much support a parent feels they receive from family, friends, and a significant other, and 3 subscale scores (with 4 items per subscale) can be calculated as well as a total score (from 12 to 84), with total scores indicating greater perceived support. If the total score and subscale scores are averaged over items, scores between 1 and 2.9 are considered “low support,” 3 to 4.9 are considered “moderate support,” and 5 to 7 are considered “high support” [34]. The MSPSS has been shown to be valid and internally reliable, with Cronbach α values ranging from .81 to .94 on individual subscales and from .83 to .92 for the total score [33].

Postpartum anxiety will be measured using the Postpartum Specific Anxiety Scale (PSAS) [35]. The PSAS is a valid and reliable instrument for assessing anxiety during the first 6 months postpartum, with Cronbach α values for individual factors ranging from .80 to .91, with an overall α value of .95 [35]. The optimal cutoff PSAS score for detecting clinical levels of anxiety is 112, with a sensitivity and specificity of 0.75 [35].

Postpartum depression will be measured using the 10-item Edinburgh Postnatal Depression Scale (EPDS) [36]. This scale can be used to screen mothers at risk for developing postpartum depression, with a score above 14 (out of 30) indicating a likelihood of having or developing postpartum depression [36]. The sensitivity of the EPDS was found to be 86% in women with depressive symptoms and 78% in women without depressive symptoms [36].

### Implementation Outcomes

To measure the implementation extent of the Essential Coaching for Every Mother program, output data available through the Twilio and TextIt platforms [30,31] will be collected per participant including, but not limited to, enrollment rate, enrollment timing (postpartum or antenatal, days postpartum), number of withdrawals, and numbers of messages received. This data collection will continue throughout the trial and will be completed after the last follow-up contacts. To measure implementation quality, in the 6-week survey women in the intervention group will be asked about user experience, perspectives on the frequency and timing of messages, and what they liked and did not like about Essential Coaching for Every Mother. Using these measures, we seek to obtain data not only on implementation extent but also on the quality of the program through open-ended questions where mothers will be asked about their experience with Essential Coaching for Every Mother in practice.

### Data Analysis

Data will be analyzed on an intention-to-treat basis (excluding women who requested to stop receiving the messages or were lost to follow-up). Demographic characteristics on maternal age, marital status, education level, and delivery method will be expressed as means and standard deviations or percentages, as applicable, based on group allocation. Postnatal contacts for themselves and their infants, health concerns, and feeding behavior will also be expressed as means and standard deviations or percentages, as applicable, based on group allocation. Any significant differences in baseline characteristics, examined through a chi-square analysis or Mann-Whitney test, will be adjusted for in the analysis. A *P* value of 0.05 will be considered significant for all outcomes.

For the primary outcome of self-efficacy, a total score will be reported using means, standard deviations, and percentages. Analysis of covariance (ANCOVA) will be used to measure whether total self-efficacy scores differ between the 2 groups, with adjustments for the pretest scores and any significant baseline characteristics.

For the first secondary objective, an ANCOVA will be used to determine whether scores differ between the 2 groups on social support, postpartum anxiety, and postpartum depression, with adjustments for the pretest scores and any significant baseline characteristics. For the second secondary objective, a 2-way ANCOVA will be used to measure whether the mean difference of self-efficacy scores differs between the 4 groups (multiparous/primiparous and intervention/control), with adjustments for any significant covariates on self-efficacy, social support, postpartum anxiety, and postpartum depression. For the third secondary objective, a 2-way repeated-measures ANCOVA will be used to explore whether changes in the key psychosocial outcome variables varied over time.

For implementation data, summative data provided by the TextIt platform and Twilio interface will be reported, and descriptive statistics will be used to describe participants’ experiences with Essential Coaching for Every Mother using means and standard deviations or percentages, as applicable. Open-ended questions from the survey will be analyzed using thematic analysis [37]. Greater understanding of the implementation extent indicators, in combination with the qualitative responses from mothers, will offer insight into the “black box” of the intervention to explore reasons for success or failure [38].

## Results

Study recruitment started on January 5, 2021, and is currently ongoing, with an anticipated date of recruitment completion of January 2022.

## Discussion

Given the lack of standardized postpartum education and support currently available, Essential Coaching for Every Mother is proposed to be an innovative solution to bridge this gap without adding a significant burden to the health care system. The goal of mHealth interventions in postnatal education is not to replace the need for in-person follow-up contacts, as these are important for health assessments of the woman and her infant by a skilled health care provider. Instead, mHealth interventions can be used to complement existing postnatal care by providing timely, standardized, relevant, and evidence-based information directly to women.

It is important to use a hybrid effectiveness-implementation design to evaluate both the Essential Coaching for Every Mother program and the process evaluation implementation to be able to offer suggestions for real-world implementation. This is the first postpartum text message program designed for women in Canada. It is expected that the findings will offer insights into whether the use of mobile technologies could be an effective strategy to ensure that research findings are effectively communicated to mothers to enhance postnatal experiences and care delivery in Nova Scotia. This strategy will also help to support the longevity of the research and may be easily transferable to other health centers and hospitals in Canada.

## Appendix

Multimedia Appendix 1 Peer-review report.

## Abbreviations

ANCOVA: analysis of covariance
CONSORT-EHEALTH: Consolidated Standards of Reporting Trials of Electronic and Mobile HEalth Applications and onLine TeleHealth
EPDS: Edinburgh Postnatal Depression Scale
KPCS: Karitane Parenting Confidence Scale
mHealth: mobile health
MSPSS: Multidimensional Scale of Perceived Social Support
PSAS: Postpartum Specific Anxiety Scale
REDCap: Research Electronic Data Capture
TIDieR: Template for Intervention Description and Replication
