# Supplementary material for: Effectiveness of a Postpartum Text Message Program (Essential Coaching for Every Mother) on Maternal Psychosocial Outcomes: Protocol for a Randomized Controlled Trial
Source: JMIR Res Protoc. 2021 Mar 25;10(3):e27138. doi: 10.2196/27138 (PMC8088838; doi:10.2196/27138)
Supplement: Multimedia Appendix 1 [file resprot_v10i3e27138_app1.pdf]

Mentored Grant
Reviewer Comments

Primary Reviewer ☐ **Secondary Reviewer** ☒

ROMEO Number: 1024260

Project Title: Essential Coaching for Every Mother: Evaluating an mHealth postnatal intervention for mothers of newborns (Phase II)

Applicant Name: Ms. Justine Dol

Purpose

This operating grant is intended to

i) support projects that will build capacity in research at the IWK, and
ii) facilitate the development of the applicant's research skills.

Evaluation of applications based on quality of the proposed project, and likelihood that the mentor/mentee relationship will lead to further skill and professional development for the mentee.

Primary Reviewer only- Briefly outline the aim(s) and description of the project (*purpose, hypothesis, approach, etc*).

Investigator Merit: (consider both the mentor and the mentee. Do they have the proper qualifications and research experience? Is the proper team in place to complete this research? Will the mentee receive adequate mentorship?)
Ms. Dol is a PhD student. She has research experience but this seems to be her first clinical research project as the primary investigator. Dr. Campbell-Yeo is an established researcher, particularly in the area of neonatal pain and uses of technology in administering health care. This seems to be her first RCT in a supervisory role.

Scientific Merit (consider impact/relevance to the IWK, clarity of goals and objectives, appropriateness of methods and analysis, challenges are identified, novelty of the research.)

Strengths: The issue being addressed by this study, lack of support for new mother, is an important problem with many implications for the health of mothers and their babies. They're proposed intervention seems novel and implementable if shown to improve outcomes. Overall the methodology for the RCT is straight forward and clear

Weaknesses: The calculated sample size of 63 per arm does not take into account drop out and loss to follow up. It seems that the coaching messages will be standardized for all participants. If messages assume women are breastfeeding then women who are formula feeding may find the messages off putting or even bothersome if they have struggled with an inability to breast feed.

Overall: A novel approach to addressing and important problem with potential for direct patient applications. The sample size needs to be expanded incorporate and expected dropout rate. The coaching messages are currently being piloted, but inclusion of these messages for review would have strengthened the application.

Budget Considerations: (budget is appropriate and properly justified) 50 hours of research assistant time for recruitment and data entry seems inadequate

Mentored Grant
Reviewer Comments

Primary Reviewer ☒ **Secondary Reviewer** ☐

ROMEO Number: 1024260

Project Title: Essential Coaching for Every Mother: Evaluating an mHealth postnatal intervention for mothers of newborns (Phase II)

Applicant Name: Dol

Purpose

This operating grant is intended to

i) support projects that will build capacity in research at the IWK, and
ii) facilitate the development of the applicant's research skills.

Evaluation of applications based on quality of the proposed project, and likelihood that the mentor/mentee relationship will lead to further skill and professional development for the mentee.

Primary Reviewer only- Briefly outline the aim(s) and description of the project (*purpose, hypothesis, approach, etc*).
The investigators propose to use mobile health to provide new first-time mothers with information during the postpartum period. Employing a 2-group parallel arm randomized controlled trial design with 1:1 allocation (n=126, 63 per arm), the team proposes to randomize first time mothers who have recently given birth to a mobile health intervention or to standard care. "Essential Coaching for Every Mother" consists of standardized text messages sent daily from birth to six-weeks postnatally that provide evidence-based information on newborn care and mother's emotional well-being. The messages have been developed and will be piloted and modified with mothers and postpartum healthcare providers prior to the study. Messages will be standardized and personalized to include babies' names. Controls will receive only the welcome message, instructions for withdrawing, and reminder messages to attend their six-week follow-up appointment three days prior to the appointment. Mothers in both groups will receive in-hospital education by a non-study nurse while on the Family Newborn Unit prior to discharge as per standard protocol at the IWK Health Centre. Evaluation measures include mothers' confidence, social support, anxiety, depression, and knowledge at baseline and six weeks. The investigators hypothesize that mothers in the intervention arm will have higher self-efficacy (primary outcome), and higher social support, lower postpartum anxiety, lower postpartum depression, and higher knowledge of newborn essential care as compared to controls (secondary outcomes).

Investigator Merit: (consider both the mentor and the mentee. Do they have the proper qualifications and research experience? Is the proper team in place to complete this research? Will the mentee receive adequate mentorship?)
The mentors have strong track records in postnatal maternal and newborn health research, with appropriate clinical and research experience. The mentee has demonstrated scholarly excellence, with a record of scholarships. The proposed project falls within the mentee's ongoing dissertation work. The team has a track record of successful collaboration, and is highly likely to successfully complete the project within the year.

Scientific Merit (consider impact/relevance to the IWK, clarity of goals and objectives, appropriateness of methods and analysis, challenges are identified, novelty of the research.)

Strengths: The proposed intervention supports an important gap in postnatal support for new mothers, is accessible, low-cost, and content will be vetted by mothers and clinicians. Supporting the postnatal health and well-being of mothers and newborns is a priority for the IWK. The study team includes mentors with strong track records. The proposal is clear, and methods/analysis are appropriate to the question under study.

Weaknesses: It is not clear whether mothers will be able to send messages (other than "stop" to end participation) or questions back to the investigators using the platform. Is the platform able to monitor and respond to messages, particularly in the event of distress? Or will participants be advised to contact a health care provider? The investigators indicate that the platform may be used to retain participants with extra messages/follow-up. They may wish to consider including the extent of follow-up as a potential confounder in their analyses.

Budget Considerations: (budget is appropriate and properly justified) It isn't clear why an RA is required for recruitment and data entry for 50 hours for online surveys ($966). $500 for open access publishing is low.
